# Sex- and weight-specific changes in the frequency of sweet treat consumption during early adolescence: a longitudinal study

**DOI:** 10.1017/S0007114521001112

**Published:** 2021-11-28

**Authors:** Sohvi Lommi, Elina Engberg, Hely Tuorila, Kaija-Leena Kolho, Heli Viljakainen

**Affiliations:** 1 Department of Public Health, University of Helsinki, Helsinki 00014, Finland; 2 Folkhälsan Research Centre, Helsinki 00250, Finland; 3 Department of Psychology and Logopedics, Faculty of Medicine, University of Helsinki, Helsinki 00014, Finland; 4 Department of Food and Nutrition, University of Helsinki, Helsinki 00014, Finland; 5 Children’s Hospital, University of Helsinki and Helsinki University Hospital, Helsinki 00014, Finland; 6 Faculty of Medicine and Health Technology, Tampere University, Tampere 33100, Finland; 7 Faculty of Medicine, University of Helsinki, Helsinki 00014, Finland

**Keywords:** Sugary products, Eating behaviours, Adolescence, Paediatric obesity, Longitudinal studies

## Abstract

The transition from childhood to adolescence is a sensitive period, triggering changes in health- and weight-related behaviours including eating habits which likely vary between girls and boys. We aimed to characterise the changes in the frequency of consumption of select sugary foods and drinks (‘sweet treats’) among 4237 Finnish girls and boys during a 2-year follow-up period. Additionally, we examined four subgroups: children whose weight or waist normalised as well as children whose weight or waist circumference increased during follow-up. An FFQ was completed at 11·1 (sd 0·9) and again at 13·4 (sd 1·1) years of age. A sum variable sweet treat index (STI, range 0–84) captured the weekly consumption frequencies of sweet treats. From baseline to follow-up, the mean STI decreased among girls from 7·1 (95 % CI 6·9, 7·3) to 6·0 (95 % CI 5·9, 6·2) (*P* < 0·001) and boys from 8·5 (95 % CI 8·3, 8·8) to 7·8 (95 % CI 7·6, 7·8) (*P* < 0·001), although both sexes increased their chocolate/sweets consumption: girls from 1·3 (95 % CI 1·3, 1·4) to 1·6 (95 % CI 1·5, 1·6) (*P* < 0·001) and boys from 1·4 (95 % CI 1·3, 1·4) to 1·6 (95 % CI 1·6, 1·7) (*P* < 0·001), and boys increased their soft drink consumption from 1·4 (95 % CI 1·3, 1·4) to 1·5 (95 % CI 1·4, 1·5) (*P* = 0·020). We found similar decreases in both the weight and waist subgroups. To conclude, the total frequency of consumption of sweet treats decreased during early adolescence. A similar trend across subgroups suggests that the frequency of consumption of sweet treats is unrelated to becoming overweight.

The transition from childhood to adolescence is a crucial period in human development, likely triggering changes in energy-related behaviours such as diet and physical activity^(1)^. During this time, adolescents gain more autonomy to make their own decisions and more often enjoy greater freedom to purchase snacks and drinks without parental supervision^(2)^. In childhood, eating habits are primarily moulded by the family environment, such as their parents’ habits, education level and socio-economic status, whilst as a teenager, peers, mass media and an individual’s own body image increasingly shape preferences, portion sizes and dieting habits^(3)^. In adolescence, food choices are increasingly characterised by less consumption of fruits and vegetables and greater consumption of soft drinks and snacks with a high-energy density such as that found in chips, candies and chocolate^(4–6)^.

Although sugar intakes have in recent decades decreased in several countries^(7)^, a high consumption of sugar-rich foods and drinks among children and adolescents raises concerns^(8)^. According to paediatric studies spanning several European countries, the consumption of sugar considerably exceeds the recommended level of 10 % energy intake^(9–11)^. Excess sugar consumption may lead to an overall low-quality diet and a positive energy balance^(12)^, increase the risk of cardiovascular diseases^(13)^ and promote weight gain^(14)^. Excess weight at a young age has reached epidemic levels, posing health risks later in life^(15–17)^. Furthermore, central obesity alone may promote risk factors for developing chronic diseases^(18)^. Alongside excess sugar consumption, adolescent diets are characterised by the insufficient intake of nutrients such as fibre, Fe and folate^(8)^.

Girls and boys reach somatic maturation at different times and tempos^(19)^. Moreover, adolescence is marked by changes in physiological requirements and increasing appetites, with boys requiring more energy than girls given greater growth in terms of height, weight and lean body mass^(20)^. Although differences in food intake may appear already during early childhood^(21)^, these changes may lead to more pronounced differences in food consumption during puberty. In general, boys exhibit unhealthier eating habits compared with girls, such as consuming more sugar-sweetened beverages and less fruit and vegetable^(6,8,22)^.

Importantly, eating habits established during childhood and adolescence are likely to track into adulthood^(23)^. Thus, adolescence, as a period of growth and change, offers an excellent time to identify potentially unhealthy eating habits to target through interventions. Since girls and boys develop at different times, it is essential to identify sex differences in changes to food consumption. Moreover, understanding food behaviour is essential when planning interventions to prevent further increases in the prevalence of adolescent obesity. The obesity-promoting role of sugar-sweetened beverages in children and adolescents is well documented^(24)^, whereas evidence regarding sugary foods and weight status remains inconclusive^(25,26)^. We previously found that less frequent sugary product consumption is associated with a higher risk of being overweight in a cross-sectional study^(27)^. Prospective studies investigating sex-specific changes in the consumption of sugary products and the relationship with weight changes, however, remain scarce within this age group. Therefore, we primarily aimed here to examine if and how the frequency of consumption of select sugary foods and drinks (‘sweet treats’) changes during the transition from childhood to adolescence in girls and boys. Secondarily, we aimed to investigate these changes in subgroups of individuals whose weight or waist normalised and among those whose weight or waist circumference increased during a 2-year follow-up period, examining girls and boys separately. Lastly, we explored changes in the consumption frequencies of other foods and drinks indicative of the overall quality of children’s diets.

## Methods

### Study design and participants

We utilised data from the Finnish Health in Teens study, a large prospective cohort consisting of more than 11 000 children – mostly aged 9–12 years at enrolment – and their guardians. Baseline data were collected from 2013 to 2014 in primary schools across Finland with no exclusion criteria, with the first active follow-up data collection beginning in 2015 through 2016. The cohort and the study protocol were described in detail elsewhere^(28)^. Here, we included 4237 children for whom information was available on sex, age, food consumption frequencies and BMI at baseline and at follow-up. The study was conducted in accordance with the Declaration of Helsinki, and the protocol was approved by the Coordinating Ethics Committee of the Hospital District of Helsinki and Uusimaa (169/13/03/00/10). Participants and one guardian per child provided their written informed consent.

### Measurements

#### FFQ

At baseline and follow-up, participants completed a sixteen-item FFQ for the preceding month, which included sweet items (chocolate/sweets, biscuits/cookies, sweet pastries, ice cream, sugary soft drinks and sugary juice drinks) and other, primarily nonsweet items (dark bread, pizza, hamburger/hot dogs, milk or buttermilk, cooked and fresh vegetables, fruits/berries, fruit juice and salty snacks as well as water (nonnutritious and omitted from further analysis)). Participants rated the frequency of their consumption of each item on a seven-point scale consisting of ‘not at all’, ‘less than once a week’, ‘once a week’, ‘2–4 times a week’, ‘5–6 times a week’, ‘once a day’ and ‘several times a day’. To better illustrate the weekly consumption frequencies, we transformed participants’ item ratings into times per week thusly: ‘not at all’ as 0, ‘less than once a week’ as 0·5 (assuming an average consumption of twice per month), ‘once a week’ as 1, ‘2–4 times a week’ as 3, ‘5–6 times a week’ as 5·5, ‘once a day’ as 7 and ‘several times a day’ as 14 times a week (assuming an average consumption of twice daily). Our FFQ was adapted from the FFQ used in the WHO’s International Health Behaviour in School-Aged Children study, which was validated and retested in Belgium and Italy among school-age children^(29,30)^.

#### Sweet treat index

Based on the FFQ, we calculated a sum score of sugary products (‘sweet treat index’ or STI, possible range 0–84) based on the weekly frequency of consumption of six foods and drinks that typically have a high sugar content and are consumed as so-called sweet treats^(27)^. Those were (1) chocolate and sweets, (2) sweet pastries, (3) biscuits/cookies, (4) ice cream, (5) sugary soft drinks and (6) sugary juice drinks.

#### Anthropometric measures

At baseline, trained fieldworkers measured participants’ height, weight and waist circumference in a standardised manner^(31)^. At follow-up, we sent the families tape measures and instructed the parents to measure and report the children’s height, weight and waist circumference. We previously found that these home measurements were sufficiently accurate for epidemiological studies^(31)^. We also calculated the BMI and categorised participants as thin, normal weight, overweight and obese according to the International Obesity Task Force age- and sex-specific guidelines^(32)^. We merged obese participants into a single category with overweight participants given the small group size (*n* 86, 2·0 % at baseline; hereafter, referred to as overweight). In addition, we created subgroups of participants who were normal weight at baseline and became overweight at follow-up (hereafter, referred to as weight gainers) and who were overweight at baseline and achieved a normal weight at follow-up (hereafter, referred to as weight normalisers). We also calculated the waist:height ratios (WtHR), an indicator of central adiposity in children and adolescents^(33)^, by dividing waist circumference by height, and categorised children into groups without central obesity (WtHR < 0·50) and with central obesity (≥0·50; missing values, *n* 21). We created subgroups of children with WtHR < 0·50 at baseline and ≥0·50 at follow-up (hereafter, referred to as waist gainers) and with WtHR ≥ 0·50 at baseline and <0·50 at follow-up (hereafter, referred to as waist normalisers).

#### Background information

We obtained maternal occupation information (as an indicator of maternal socio-economic status) at the time of child’s birth from the Medical Birth Register from the National Institute for Health and Welfare (THL)^(34)^. Mothers were categorised as upper-level employees, lower-level employees, manual workers, students and other (including self-employed persons, stay-at-home mothers, unemployed persons and pensioners).

### Statistical analyses

To eliminate outliers, we computed the *z*-scores for STI at baseline and at follow-up and removed cases with a standard deviation (sd) of +3 (*n* 157; there were no cases with a –3 sd). The *χ*^2^ test was used to examine the differences for categorical background characteristics between sexes. For a variable with more than two groups, we compared column proportions using the *z*-test after finding a significant difference between the sexes in the *χ*^2^ test. We used the independent samples *t* test to examine the differences in continuous background characteristics comparing girls and boys. In addition, we used the paired samples *t* test to examine longitudinal changes in STI and the frequencies of consumption for all FFQ items separately among girls and boys. To determine if girls and boys changed their behaviours in different ways, we examined the interaction of sex and time using a two-way mixed analysis of covariance adjusting for age at baseline and follow-up time. We repeated the analyses for subgroups of weight normalisers, weight gainers, waist normalisers and waist gainers. Results are presented as crude means and standard deviations or CIs. Moreover, we conducted a sensitivity analysis comparing the present sample with the rest of the cohort (comprising children who either did not participate in the follow-up or had missing values in age, sex, BMI or FFQ items; *n* 7170). The number of missing values is reported in tables and figures, and they are not included in the analysis. We used IBM’s SPSS Statistics software program, version 25 (IBM Corp.) for all statistical analyses and set the level of statistical significance to *P* < 0·05.

## Results

### Participant characteristics

The mean (sd) age of participants was 11·1 (sd 0·9) years at baseline and 13·4 (sd 1·1) years at follow-up. The mean follow-up time was 2·3 (sd 0·3) years. These did not differ between girls and boys (*P* = 0·274; *P* = 0·356 and *P* = 0·971, respectively). Table 1 summarises other participant characteristics. We detected no difference between BMI groups at follow-up or based on maternal socio-economic status when comparing girls and boys. At baseline, a larger proportion of girls than boys were thin. Both at baseline and at follow-up, a higher proportion of boys than girls exhibited central obesity (WtHR ≥ 0·50), with this difference increasing at follow-up. Among girls, 99 (4·4 %) were weight normalisers and 105 (4·6 %) weight gainers, whilst 57 (2·5 %) were waist normalisers and 81 (3·6 %) waist gainers. Among boys, the corresponding figures were 63 (3·2 %), 88 (4·5 %), 53 (2·7 %) and 118 (6·0 %), respectively. Moreover, according to the sensitivity analysis (online Supplementary Table S1), the present sample exhibited a lower proportion of participants with overweight and central obesity, and a larger proportion of participants with maternal upper-level employment compared with the participants who did not complete the follow-up or were excluded based on missing values (*P* < 0·001 for all). Moreover, they demonstrated a lower mean STI (7·8 (sd 5·4) *v*. 9·8 (sd 9·3), *P* < 0·001).

**Table 1.** Participant characteristics (*n* 4237) by sex (Numbers and percentages)

	Girls (*n* 2271)		Boys (*n* 1966)		
	*n*	%	*n*	%	*P* *
Weight status† at baseline					
Thin‡	320	14·1	195	9·9	<0·001
Normal weight‡	1646	72·5	1508	76·7	
Overweight	305	13·4	263	13·4	
Weight status† at follow-up					
Thin	185	8·1	150	7·6	0·588
Normal weight	1775	78·2	1528	77·7	
Overweight	311	13·7	288	14·6	
Central obesity§ at baseline					
No	2114	93·1	1779	90·5	0·001
Yes	151	6·6	183	9·3	
Data missing||	6	0·3	4	0·2	
Central obesity§ at follow-up					
No	2093	92·2	1715	87·2	<0·001
Yes	173	7·6	245	12·5	
Data missing||	5	0·2	6	0·3	
Maternal SES¶					
Upper-level employees	741	32·6	657	33·4	0·387
Lower-level employees	836	36·8	759	38·6	
Manual workers	226	10·0	166	8·4	
Students	212	9·3	171	8·7	
Other	149	6·6	130	6·6	
Data missing||	107	4·7	83	4·2	

* Results from a *χ*^2^ test.

† BMI categorised based on the International Obesity Task Force (IOTF) age- and sex-specific guidelines^(32)^. ‘Overweight’ includes obese individuals.

‡ Statistically significant difference in column proportions. Results from the *z*-test.

§ Central obesity estimated as waist:height ratio (WtHR) and categorised as no (WtHR < 0·50) or yes (WtHR ≥ 0·50).

|| Missing data not included in the analysis.

¶ Maternal occupation at the time of child’s birth as an indicator of socio-economic status (SES) from the Medical Birth Register from the National Institute for Health and Welfare (THL)^(34)^.

**Table 2.** Change in weekly consumption frequencies for other FFQ items during a 2-year follow-up period among girls and boys (Mean values and standard deviations, *n* 4237)

	Girls (*n* 2271)						Boys (*n* 1966)						
	Baseline		Follow-up				Baseline		Follow-up				Sex × time
	Mean	sd	Mean	sd	*P* *	Change +/–	Mean	sd	Mean	sd	*P* *	Change +/–	*P* †
Dark bread	5·0	4·1	5·3	4·3	0·029	+	5·5	4·3	5·4	4·4	0·353	0	0·029
Pizza	0·5	0·7	0·4	0·3	0·009	–	0·6	0·8	0·5	0·4	<0·001	–	0·106
Hamburgers or hot dogs	0·4	0·6	0·4	0·5	0·002	–	0·6	0·8	0·5	0·5	0·001	–	0·465
Milk or buttermilk	11·0	4·7	10·2	5·2	<0·001	–	11·1	4·7	11·4	4·5	<0·001	+	0·007
Cooked vegetables	2·5	2·9	2·6	2·8	0·418	0	2·4	2·8	2·2	2·4	0·003	–	0·003
Fresh vegetables	6·6	4·4	7·4	4·4	<0·001	+	5·9	4·3	6·2	4·1	0·003	+	<0·001
Fruits and berries	5·8	4·3	6·0	4·3	0·125	0	4·9	4·0	4·4	3·6	<0·001	–	<0·001
Fresh juice	3·6	3·7	2·4	3·0	<0·001	–	3·8	3·8	2·6	3·1	<0·001	–	0·967
Salty snacks	0·9	1·0	0·8	0·7	0·007	–	1·0	1·1	0·9	0·8	0·005	–	0·708

* Results from a paired samples *t* test.

† Results from a two-way mixed ANCOVA (interaction sex × time), adjusted for age at baseline and follow-up time period.

### Changes in sweet treat consumption

**Fig. 1:**
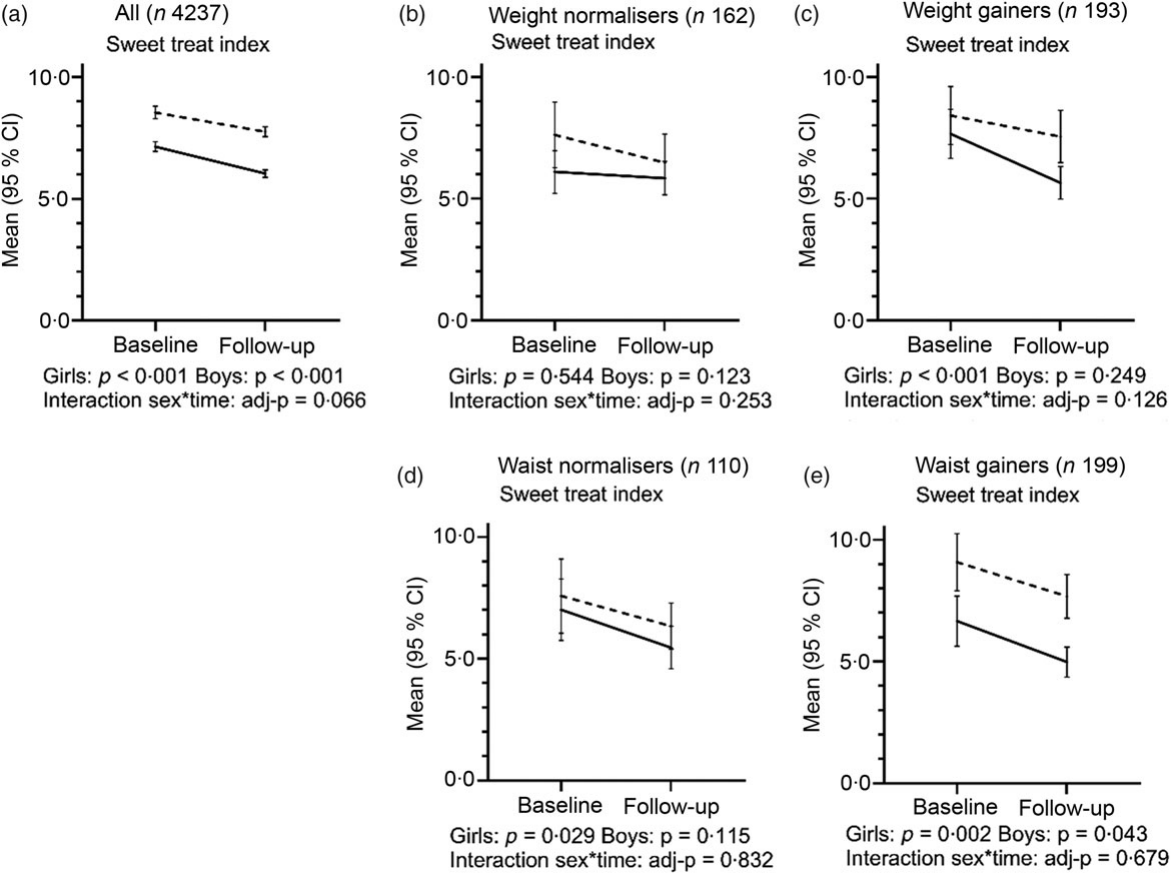
Change in sweet treat index (sum of weekly consumption frequencies of sweet treats) during a 2-year follow-up period in girls and boys among (a) the entire sample, (b) weight normalisers (from overweight to normal weight), (c) weight gainers (from normal weight to overweight), (d) waist normalisers (from WtHR ≥ 0·50 to < 0·50) and (e) waist gainers (from WtHR < 0·50 to ≥ 0·50). Results for comparisons within sex from a paired samples *t* test. Results for comparisons between sexes (interaction for sex × time) from a two-way mixed ANCOVA, adjusted for age at baseline and follow-up time. a) Girls (*n* 2271); Boys (*n* 1966)); b) Girls (*n* 99); Boys (*n* 63); c) Girls (*n* 105); Boys (*n* 88); d) Girls (*n* 57); Boys (*n* 53); e) Girls (*n* 81); Boys (*n* 118)
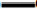
Girls 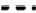
Boys.

Boys exhibited a higher STI compared with girls at baseline and at follow-up. STI declined among both girls and boys across the entire sample (Fig. 1(a)). We also observed a decrease in STI among subgroups of girls vis-à-vis weight gain and waist normalising and among girls and boys in the waist gain groups (Fig. 1(c)–(e)). Among girls who exhibited a normalising weight and among boys who gained weight or exhibited a normalising waist, we also detected a declining trend, although it was not statistically significant (Fig. 1(b)–(d)).

**Fig. 2:**
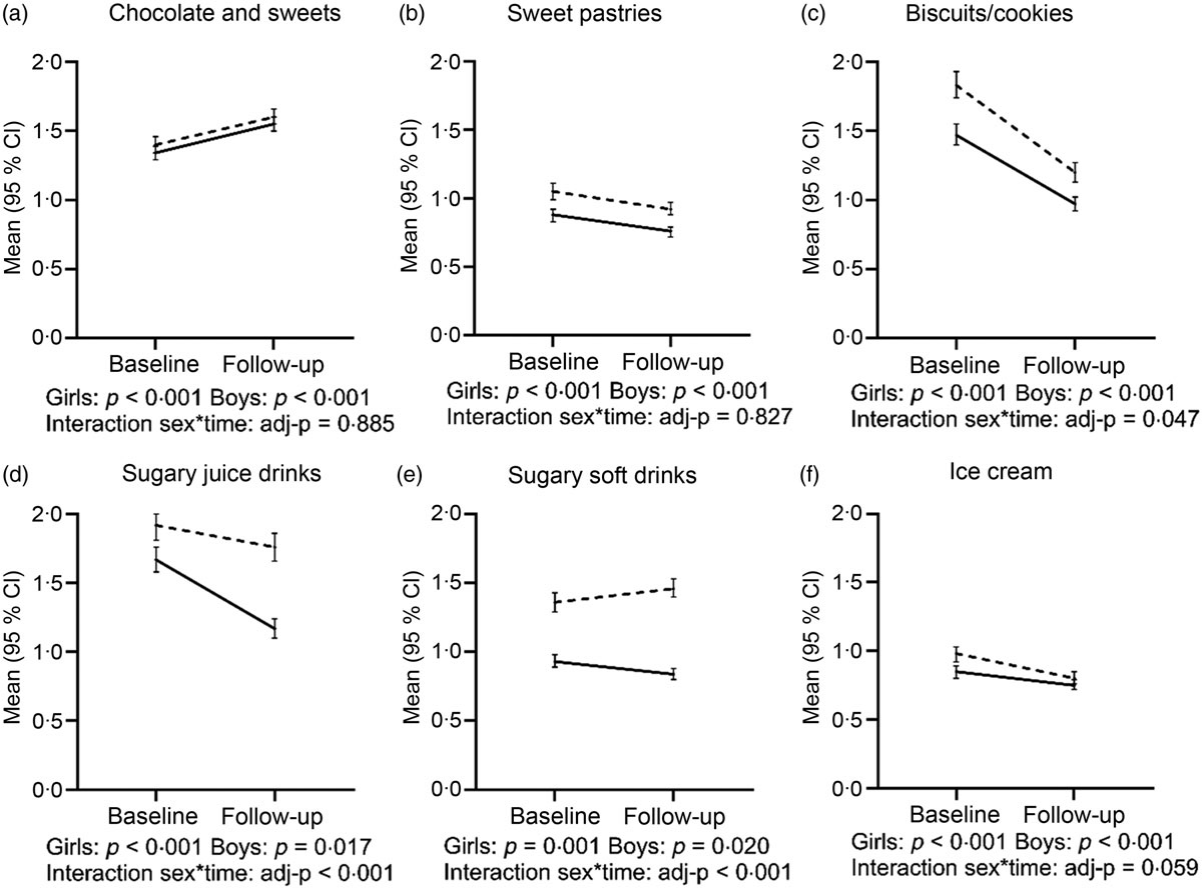
Change in weekly consumption frequencies for individual sweet treat items during a 2-year follow-up period among girls and boys (*n* 4237). Results for within-sex comparisons from a paired samples *t* test. Results for comparisons between sexes (interaction for sex × time) from a two-way mixed ANCOVA, adjusted for age at baseline and follow-up time. 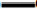 Girls (*n* 2271); 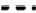 Boys (*n* 1966).

We examined separately changes in the consumption frequencies for individual STI items during the 2-year follow-up period among girls and boys (Fig. 2). Briefly, we found that the consumption of chocolate and sweets increased, whilst consumption of sweet pastries and ice cream decreased among both sexes in a similar manner (no interaction for sex with time). By contrast, the consumption of sugary soft drinks decreased among girls, but increased among boys (interaction between sex and time, *P* < 0·001). Moreover, both sexes decreased their consumption of sugary juice drinks and biscuits/cookies. However, the decreasing consumption of sugary juice drinks appeared steeper among girls compared with boys, whilst that for biscuits/cookies was steeper among boys than girls (interaction between sex and time, *P* < 0·001 and *P* = 0·047, respectively).

Online Supplementary Tables S2–S5 summarise the changes to the consumption frequencies for individual STI items within subgroups. These results reveal a similar trend as across the entire cohort or indicate no statistically significant differences. Boys exhibiting a normalising weight decreased their consumption of biscuits/cookies (online Supplementary Table S2). Girls who gained weight decreased their frequency of consumption of biscuits/cookies and sugary juice drinks, whilst boys increased their consumption of chocolate and sweets and decreased their consumption of biscuits/cookies (online Supplementary Table S3). Waist normalising girls and boys decreased their consumption of biscuits/cookies, whereas boys in this group decreased their sweet pastry consumption (online Supplementary Table S4). Among the subgroup of waist gainers (online Supplementary Table S5), girls and boys decreased their consumption of biscuits/cookies, whilst boys decreased their consumption of sweet pastries.

### Changes in other food consumption

We also investigated how the consumption of other FFQ items changed during the 2-year follow-up period (Table 2). The consumption of dark bread increased among girls, whereas we detected no change among boys (interaction between sex and time, *P* = 0·029). Moreover, the consumption of pizza, hamburgers or hot dogs, fresh juice and salty snacks decreased among girls and boys (no interaction between sex and time), whilst the consumption of milk decreased among girls and increased among boys (interaction between sex and time, *P* = 0·007). We also observed an increase in the consumption of fresh vegetables among both girls and boys. Yet, we found no change in the consumption of cooked vegetables and fruits and berries among girls, whereas among boys, consumption increased (interaction between sex and time, *P* = 0·003 and *P* < 0·001, respectively).

Changes in the consumption frequencies of other FFQ items in the subgroups appear in online Supplementary Tables S2–S5. These results reveal a similar trend as across the entire cohort or indicate no statistically significant differences. Weight normalising boys decreased their consumption of fresh juice (online Supplementary Table S2). Weight gaining girls decreased their consumption of milk or buttermilk, fresh juice and salty snacks, whilst boys decreased their consumption of fruits and berries and fresh juice (online Supplementary Table S3). Among waist normalisers and gainers, girls decreased and boys increased their consumption of milk or buttermilk (interaction between sex and time, *P* < 0·001 for both interactions; online Supplementary Tables S4 and S5). Moreover, waist gaining boys increased their consumption of fresh vegetables, but decreased their consumption of fruits and berries and fresh juice.

## Discussion

This study aimed to characterise the temporal development of select sweet treat and nonsweet food consumption during the transitory period from childhood to adolescence among more than 4000 Finnish children and to identify differences between girls and boys. Our primary finding indicates that the overall frequency of consumption of sugary, sweet foods and drinks (expressed as STI) decreased during the follow-up period among girls and boys across our sample. However, examining individual STI items, girls and boys increased their consumption of chocolate and sweets, and boys – but not girls – increased their consumption of sugary soft drinks. Furthermore, in the subgroups consisting of weight and waist normalisers and weight and waist gainers, we observed similar declining trends for total sweet treat consumption. To our knowledge, this study is the first one to utilise a sum score of sweet treats in a prospective study setting to illustrate a dietary behaviour of sweet treat consumption, how it changes and its associations with weight change.

A decreasing STI suggests a positive development in the dietary behaviour of adolescents, which agrees with previous studies in which a decrease in the intake of added sugars has been shown during adolescence and as adolescents transition to adulthood^(35,36)^. Mirroring our results, South African girls and boys increased their consumption of chocolate and decreased their consumption of ice cream^(37)^, although unlike our findings decreased their consumption of sweets. Studies from Sweden and Germany detected no change in the consumption of sweet foods from childhood to adolescence^(38,39)^. Agreeing with our results, intake of free sugars decreased among German children and adolescents from the age of 6–10 years through to 15–18 years^(40)^. However, intake of free sugars from sugar-sweetened beverages (including a wide range of sugary drinks) increased in girls and boys. Interestingly, a key difference between the sexes in our sample was the increase in sugary soft drink consumption among boys specifically. This agreed with other studies, such as studies with a 5-year follow-up period, in which 10-year-old German boys increased their consumption of energetic drinks^(39)^ and in which 13-year-old US boys increased their soda and sugar-sweetened beverage consumption^(22)^, whereas girls exhibited no significant changes. However, these studies reported other drinks in addition to soft drinks. In contrast, a study among 13-year-old South African girls and boys found an increase in soft drink consumption during a 5-year follow-up among both sexes^(37)^. Cultural differences, such as better availability and different consumption patterns in low- and middle-income compared with high-income countries^(37)^, may play a role in soft drink consumption, thereby explaining the different changes in girls’ behaviours. The differences we observed between girls and boys may reflect the higher energy needs of boys, which manifest during adolescence. Boys have reported to consume more beverages compared with girls^(6,^ ^41^^)^. Moreover, girls may have substituted sugary soft drinks for diet soft drinks. However, we did not collect information on diet soft drink consumption.

The declining STI among subgroups suggests that the frequency of consumption of sweet treats is not a relevant factor in weight development among this age group. Evidence regarding added sugars and the risk for obesity has remained inconsistent^(13)^. Yet, increasing sugar intake, primarily in the form of sugar-sweetened beverages, has been shown to associate with weight gain, while decreasing such intake is associated with weight loss^(14)^. Our results, however, do not support this, since we observed a decrease in sugary juice drink consumption among girls exhibiting a weight gain. Supporting our finding, snack food intake (including both sugary and savoury foods) did not associate with weight gain among US adolescents^(42,43)^. In the cross-sectional study^(27)^, we showed that being in the lowest quartile of sweet treat consumption frequencies is associated with an increased risk of being overweight. The association may be explained by overweight children restricting their sweet treat consumption^(27)^. Here, we speculate that adolescents who became overweight or centrally obese may have restricted their sweet treat consumption frequencies either as an attempt to control or reverse the weight gain or central obesity. However, underreporting may be more common for foods and drinks considered unhealthy and among overweight and obese individuals and may increase with age^(44)^. Furthermore, we cannot exclude the possibility that our FFQ measuring only frequencies is insufficiently accurate to detect dietary changes related to weight gain.

In addition, we identified other positive changes in food consumption among both sexes, such as decreasing frequencies in the consumption of pizza, hamburgers or hot dogs and salty snacks. Similarly, among 16-year-old US adolescents, the consumption of salty snacks decreased among girls and boys, with girls also eating less fast food over a 2-year follow-up period^(45)^. In contrast, another study found that 13-year-old US adolescents increased their fast food consumption^(46)^, as did 16-year-old adolescents^(47)^ during a 5-year follow-up period. Taken together, these findings imply that an increase in fast food consumption may occur at a later point during adolescence than we examined in our sample.

Another positive change was the increase in consumption of fresh vegetables among girls and boys. Additionally, boys increased their consumption of cooked vegetables. Typically, fruit and vegetable consumption decreases during adolescence^(48)^. Thus, we witnessed an encouraging development within our sample. However, boys decreased their fruit consumption, a trend also observed among 10-year-old German boys during a 5-year follow-up period^(39)^, highlighting the need to focus on encouraging fruit consumption among boys specifically. In contrast, we also found that the consumption of fresh juice, a nutrient-rich drink, decreased among girls and boys. The literature provides mixed observations regarding the consumption of fresh juice, with increases reported among US adolescents^(4)^ and decreases among German adolescents^(49)^. We observed an increase in consumption of dark bread (in Finland, typically, rye bread) among girls – a promising result given that adolescents’ fibre intake is lower than the recommended level^(8)^. Simultaneously, girls decreased their consumption of milk and buttermilk, whereas boys increased their consumption. A decrease in dairy product consumption was observed among 14-year-old Australian adolescents over a 3-year period, possibly carrying adverse health effects^(50)^. However, we lack information about the consumption of other milk products, such as yogurt and, thus, cannot conclude whether this decrease among girls may adversely affect them. Yet, again, the increase observed among boys could indicate their higher energy needs compared with girls.

A sweet taste carries a strong hedonistic appeal^(51)^, and children have an innate preference for sweet items, which, over time, decreases^(52)^. Children’s stronger preference for sweetness compared with adults may reflect their energy needs during growth^(53)^. The change in sweetness sensitivity towards adult-like preferences may occur during adolescence^(54)^. However, this is unlikely to explain our results. More probable explanations for the changes we observed stem from environmental factors likely influencing adolescent eating behaviours, such as influence from peers, food availability, convenience and mass media messaging^(20)^. Biological drivers, such as the gustatory function^(55)^, may play a role alongside psychological and social factors^(21)^, thus explaining the sex differences we observed. For example, 4- to 16-year-old boys reported liking fatty and sugary foods more than girls, whereas girls reported a preference for fruits and vegetables^(56)^. In contrast, young adult Finnish women report a stronger preference for sweet products than young Finnish men^(57)^. Moreover, parenting style affects child’s food intake and weight-related behaviours such as consumption of fruit and vegetables and sugar-sweetened beverages, possibly in a different manner in girls and boys^(58–60)^.

This study carries several potential limitations. The self-administered FFQ as used represents a crude measure of habitual diet and does not include portion sizes. Whilst this allows us to detect changes in consumption frequencies, we cannot measure absolute intake nor assess the level of misreporting. We also lacked information on energy drink consumption, possibly relevant to this specific age group. The FFQ – adapted from the WHO Health Behaviour in School-Age Children study’s validated and retested^(29,30)^ FFQ – was not validated, but in general, short FFQ measuring a short time span, not assessing portion sizes and completed by child/adolescent (instead of parent), have been found with the highest validity^(61)^. Moreover, we had to rely on age as an indicator of puberty since we lacked specific information on participants’ puberty status. However, we took into account the different maturation time between the sexes by examining girls and boys separately and by adjusting our analysis for age at baseline and follow-up time. We acknowledge that, due to the relatively short follow-up time period, the age ranges at baseline and follow-up overlap, possibly impacting our analysis and results (such as revealing no changes). Despite this, we observed positive changes in food consumption. Moreover, adjusting appeared to marginally impact the means and 95 % confidence intervals (data not shown). Specifically, the lower proportion of children with overweight and the higher proportion of mothers with upper-level employment in the Finnish Health in Teens cohort compared with the general Finnish population^(27)^ suggest that our sample may be more health conscious than the entire population; thus, our findings on sweet treat consumption may not apply to children from lower socio-economic backgrounds. Moreover, participants with higher parental education may be more likely to continue through to follow-up^(28)^. Indeed, lost to follow-up is common in longitudinal studies, potentially creating a risk of selection bias. In accordance, the sensitivity analysis revealed that maternal upper-level employment was more common among the present sample compared with children who did not participate in the follow-up or were otherwise excluded. Further analyses are needed to address sweet treat consumption among children from lower socio-economic backgrounds. Moreover, the subgroup analysis may be underpowered due to small group sizes leading to the possibility of type II error. The analysis should be repeated with a larger sample size.

Despite these limitations, this study adds to the existing body of knowledge regarding the temporal changes in adolescent eating behaviour. The strengths of our study include its large, well-defined sample of adolescents across Finland and the longitudinal study setting allowing us to observe patterns over time. In addition to BMI, we included WtHR as a measure of excess weight, since a positive energy balance can be initially observed as an increase in waist circumference. Despite some of the abovementioned shortcomings accompanying the use of a short FFQ, our instrument measured habitual consumption rather than a 24-h dietary recall and proved simple and quick to complete as a part of longer surveys. Such a short measure could be feasible in clinical settings, such as school health care, if proven to show relevant associations between behaviour and health outcomes. Furthermore, trained fieldworkers measured weight, height and waist circumference in a standardised manner at baseline, whilst follow-up measurements, despite being self-reported, also proved valid^(31)^.

To conclude, our results indicate a modest and primarily positive development in eating habits among 11-year-old adolescents during a 2-year follow-up period. However, undesirable changes, such as an increasing consumption of chocolate and sweets among girls and boys and an increased soft drink consumption among boys, require further action. This study highlights the differences in food consumption between girls and boys and helps to target dietary interventions. Furthermore, our findings expand the limited understanding of food consumption behaviours during the important developmental period of early adolescence and among adolescents who gain or lose excess weight. Further studies with precise dietary data are needed in order to substantiate our findings.

## Supplementary material

For supplementary material accompanying this paper visit https://doi.org/10.1017/S0007114521001112. click here to view supplementary material
