# Supplementary material for: Sex- and weight-specific changes in the frequency of sweet treat consumption during early adolescence: a longitudinal study
Source: Br J Nutr. 2021 Mar 31;126(10):1592–600. doi: 10.1017/S0007114521001112 (PMC8524426; doi:10.1017/S0007114521001112)
Supplement: Supplementary file 1 [file S0007114521001112sup001.docx]

Supplementary Table 1. Comparison of participant characteristics and sweet treat index

between children in the present study (‘included’) and the rest of the cohort (‘excluded’) at baseline.

|  | **Included (n=4237)** | **Excluded^a^ (n=7170)** | **p** |
| --- | --- | --- | --- |
| **Sex, n (%)** |  |  |  |
| girl | 2271 (53.6) | 3710 (51.8) | 0.058^b^ |
| boy | 1966 (46.4) | 3457 (48.2) |  |
| data missing, n | 0 | 3 |  |
| **Weight status^c^, n (%)** | |  |  |
| thin | 515 (12.2) | 660 (10.3) | <0.001^b^ |
| normal weight | 3154 (74.4) | 4700 (73.3) |  |
| overweight | 568 (13.4) | 1049 (16.4) |  |
| data missing, n | 0 | 761 |  |
| **Central obesity^d^, n (%)** | |  |  |
| no | 3893 (92.1) | 5757 (88.8) | <0.001^b^ |
| yes | 334 (7.9) | 729 (11.2) |  |
| data missing, n | 10 | 684 |  |
| **Maternal SES^e^, n (%)** | |  |  |
| upper-level | 1398 (34.5) | 1771 (26.7) | <0.001^b^ |
| lower-level | 1595 (39.4) | 2705 (40.8) |  |
| manual workers | 392 (9.7) | 887 (13.4) |  |
| students | 383 (9.5) | 744 (11.2) |  |
| other | 279 (6.9) | 524 (7.9) |  |
| data missing, n | 190 | 539 |  |
| **Age, mean (SD)** | 11.1 (0.9) | 11.2 (0.8) | <0.001^f^ |
| data missing, n | 0 | 95 |  |
| **Sweet treat index, mean (SD)** | 7.8 (5.4) | 9.8 (9.3) | <0.001^f^ |
| data missing, n | 0 | 2063 |  |

^a^ ‘Excluded’ comprises children who did not participate in the follow-up or were excluded from the present study due to missing values in age, sex, BMI or food consumption frequencies.

^b^Results from a chi-square test.

^c^Body mass index (BMI) categorised based on the International Obesity Task Force (IOTF) age- and sex-specific guidelines^(29)^. ‘Overweight’ includes obese individuals.

^d^Central obesity estimated as waist–height ratio (WtHR) and categorised as no (WtHR < 0.50) or yes (WtHR ≥ 0.50).

^e^Maternal occupation at the time of child’s birth as an indicator of socioeconomic status (SES) from the Medical Birth Register from the National Institute for Health and Welfare (THL)^(31)^.

^f^Results from an independent samples t-test.

Supplementary Table 2. Mean (SD) change in weekly consumption frequencies of STI items and other FFQ items among weight normalisers (girls and boys who were overweight at baseline and normal weight at follow-up; n = 162).

|  | **Girls (n = 99)** | | | | | | **Boys (n = 63)** | | | | | | |  |
| --- | --- | --- | --- | --- | --- | --- | --- | --- | --- | --- | --- | --- | --- | --- |
|  | **Baseline** | | **Follow-up** | |  |  | **Baseline** | | **Follow-up** | |  | |  | **Sex*time** |
|  | **Mean (±SD)** | | **Mean (±SD)** | | **p*^a^*** | **Change +/-** | **Mean (±SD)** | | **Mean (±SD)** | | **p*^a^*** | | **Change +/-** | **p*^b^*** |
| **STI item** | | |  |  |  |  |  |  |  |  |  | |  |  |
| Chocolate and sweets | 1.1 | (1.0) | 1.3 | (1.0) | 0.213 | 0 | 1.1 | (0.7) | 1.2 | (1.0) | 0.509 | | 0 | 0.711 |
| Sweet pastries | 0.7 | (0.8) | 0.7 | (0.7) | 0.776 | 0 | 1.0 | (1.3) | 0.7 | (0.6) | 0.066 | | 0 | 0.068 |
| Biscuits/cookies | 1.0 | (1.6) | 1.1 | (1.8) | 0.943 | 0 | 1.7 | (2.0) | 1.0 | (1.4) | 0.004 | | – | 0.007 |
| Sugary juice drinks | 1.4 | (1.7) | 1.1 | (1.5) | 0.258 | 0 | 1.8 | (2.1) | 1.5 | (2.2) | 0.279 | | 0 | 0.809 |
| Sugary soft drinks | 1.0 | (1.7) | 0.9 | (0.8) | 0.561 | 0 | 1.3 | (1.3) | 1.5 | (2.0) | 0.372 | | 0 | 0.260 |
| Ice cream | 0.9 | (1.2) | 0.8 | (0.9) | 0.369 | 0 | 0.8 | (1.0) | 0.7 | (0.7) | 0.222 | | 0 | 0.771 |
| **Other FFQ item** |  |  |  |  |  |  |  |  |  |  | |  |  |  |
| Dark bread | 5.3 | (3.7) | 5.4 | (4.2) | 0.758 | 0 | 4.7 | (3.9) | 4.8 | (4.2) | 0.874 | | 0 | 0.858 |
| Pizza | 0.5 | (0.8) | 0.4 | (0.3) | 0.117 | 0 | 0.6 | (0.8) | 0.5 | (0.3) | 0.089 | | 0 | 0.693 |
| Hamburgers or hot dogs | 0.3 | (0.2) | 0.3 | (0.3) | 0.540 | 0 | 0.5 | (0.4) | 0.5 | (0.5) | 0.899 | | 0 | 0.682 |
| Milk | 10.5 | (4.7) | 9.6 | (5.3) | 0.094 | 0 | 10.4 | (5.2) | 11.2 | (4.9) | 0.171 | | 0 | 0.018 |
| Cooked vegetables | 2.5 | (2.8) | 2.7 | (2.7) | 0.598 | 0 | 2.6 | (3.2) | 2.2 | (2.4) | 0.328 | | 0 | 0.373 |
| Fresh vegetables | 6.5 | (4.3) | 7.1 | (4.0) | 0.280 | 0 | 6.0 | (4.5) | 5.4 | (3.9) | 0.245 | | 0 | 0.169 |
| Fruits and berries | 5.6 | (4.4) | 6.0 | (4.4) | 0.383 | 0 | 4.5 | (4.1) | 4.2 | (3.9) | 0.507 | | 0 | 0.344 |
| Fresh juice | 3.2 | (3.3) | 2.7 | (3.6) | 0.265 | 0 | 3.9 | (3.9) | 2.4 | (2.6) | 0.003 | | – | 0.219 |
| Salty snacks | 0.8 | (0.9) | 0.8 | (0.7) | 1.000 | 0 | 0.9 | (0.8) | 0.8 | (0.8) | 0.736 | | 0 | 0.665 |

^a^Results from a paired samples t-test.

^b^Results from a two-way mixed ANCOVA (interaction for sex*time), adjusted for age at baseline and follow-up time.

Abbreviations: STI, sweet treat index; FFQ, food frequency questionnaire; SD, standard deviation.

Supplementary Table 3. Mean (SD) change in weekly consumption frequencies of STI items and other FFQ items among weight gainers (girls and boys who were normal weight at baseline and overweight at follow-up; n = 193).

|  | **Girls (n = 105)** | | | | | | **Boys (n = 88)** | | | | | |  |
| --- | --- | --- | --- | --- | --- | --- | --- | --- | --- | --- | --- | --- | --- |
|  | **Baseline** | | **Follow-up** | |  |  | **Baseline** | | **Follow-up** | |  |  | **Sex*time** |
|  | **Mean (±SD)** | | **Mean (±SD)** | | **p*^a^*** | **Change +/-** | **Mean (±SD)** | | **Mean (±SD)** | | **p*^a^*** | **Change +/-** | **p*^b^*** |
| **STI item** |  |  |  |  |  |  |  |  |  |  |  |  |  |
| Chocolate and sweets | 1.4 | (1.6) | 1.4 | (1.0) | 0.956 | 0 | 1.2 | (1.0) | 1.6 | (1.0) | 0.047 | + | 0.182 |
| Sweet pastries | 0.8 | (0.8) | 0.7 | (0.7) | 0.229 | 0 | 0.9 | (1.1) | 0.8 | (0.9) | 0.472 | 0 | 0.975 |
| Biscuits/cookies | 1.7 | (2.2) | 0.8 | (0.9) | <0.001 | – | 1.9 | (1.9) | 1.2 | (2.0) | 0.008 | – | 0.459 |
| Sugary juice drinks | 1.9 | (2.2) | 1.2 | (1.6) | 0.007 | – | 1.9 | (2.2) | 1.7 | (2.6) | 0.656 | 0 | 0.108 |
| Sugary soft drinks | 1.1 | (1.2) | 0.8 | (1.0) | 0.067 | 0 | 1.6 | (1.7) | 1.7 | (1.6) | 0.724 | 0 | 0.159 |
| Ice cream | 0.8 | (1.1) | 0.8 | (0.9) | 0.683 | 0 | 0.9 | (1.2) | 0.6 | (0.6) | 0.056 | 0 | 0.191 |
| **Other FFQ item** |  |  |  |  |  |  |  |  |  |  |  |  |  |
| Dark bread | 4.9 | (4.0) | 4.7 | (3.8) | 0.700 | 0 | 5.6 | (4.4) | 5.2 | (4.2) | 0.362 | 0 | 0.719 |
| Pizza | 0.5 | (0.8) | 0.5 | (0.3) | 0.361 | 0 | 0.7 | (1.1) | 0.5 | (0.5) | 0.265 | 0 | 0.688 |
| Hamburgers or hot dogs | 0.4 | (0.4) | 0.6 | (1.4) | 0.189 | 0 | 0.6 | (0.8) | 0.5 | (0.4) | 0.282 | 0 | 0.106 |
| Milk or buttermilk | 10.5 | (5.3) | 9.0 | (5.8) | 0.003 | – | 11.3 | (4.8) | 10.7 | (5.2) | 0.177 | 0 | 0.259 |
| Cooked vegetables | 3.1 | (3.3) | 2.9 | (3.0) | 0.719 | 0 | 2.4 | (2.3) | 2.2 | (2.5) | 0.639 | 0 | 0.874 |
| Fresh vegetables | 7.2 | (4.5) | 7.7 | (4.7) | 0.364 | 0 | 5.2 | (4.1) | 5.7 | (3.9) | 0.270 | 0 | 0.899 |
| Fruits and berries | 6.0 | (4.5) | 5.9 | (4.6) | 0.789 | 0 | 4.8 | (4.0) | 3.6 | (3.4) | 0.011 | – | 0.151 |
| Fresh juice | 3.6 | (3.7) | 2.1 | (2.5) | <0.001 | – | 3.5 | (3.5) | 2.4 | (3.0) | 0.013 | – | 0.379 |
| Salty snacks | 1.2 | (1.7) | 0.8 | (0.7) | 0.028 | – | 0.9 | (0.8) | 0.9 | (0.7) | 0.869 | 0 | 0.090 |

^a^Results from a paired samples t-test.

^b^Results from a two-way mixed ANCOVA (interaction for sex*time), adjusted for age at baseline and follow-up time.

Abbreviations: STI, sweet treat index; FFQ, food frequency questionnaire; SD, standard deviation.

Supplementary Table 4. Mean (SD) change in weekly consumption frequencies of STI items and other FFQ items among waist normalisers (girls and boys whose WtHR was ≥ 0.50 at baseline and < 0.50 at follow-up; n = 110). Missing values for WtHR n = 21.

|  | **Girls (n = 57)** | | | | | | **Boys (n = 53)** | | | | | |  |
| --- | --- | --- | --- | --- | --- | --- | --- | --- | --- | --- | --- | --- | --- |
|  | **Baseline** | | **Follow-up** | |  |  | **Baseline** | | **Follow-up** | |  |  | **Sex*time** |
|  | **Mean (±SD)** | | **Mean (±SD)** | | **p*^a^*** | **Change +/-** | **Mean**  **(±SD)** | | **Mean (±SD)** | | **p*^a^*** | **Change +/-** | **p*^b^*** |
| **STI item** | | |  |  |  |  |  |  |  |  |  |  |  |
| Chocolate and sweets | 1.3 | (1.2) | 1.2 | (0.9) | 0.514 | 0 | 1.3 | (1.2) | 1.2 | (0.9) | 0.585 | 0 | 0.714 |
| Sweet pastries | 0.8 | (1.0) | 0.7 | (0.7) | 0.485 | 0 | 0.9 | (1.3) | 0.8 | (0.9) | 0.530 | 0 | 0.943 |
| Biscuits/cookies | 1.4 | (2.1) | 0.8 | (1.1) | 0.037 | – | 1.5 | (1.8) | 1.0 | (1.2) | 0.044 | – | 0.749 |
| Sugary juice drinks | 1.5 | (1.8) | 1.2 | (1.6) | 0.197 | 0 | 1.7 | (2.9) | 1.5 | (1.6) | 0.615 | 0 | 0.864 |
| Sugary soft drinks | 1.1 | (1.9) | 0.9 | (0.9) | 0.603 | 0 | 1.4 | (2.2) | 1.3 | (1.2) | 0.715 | 0 | 0.884 |
| Ice cream | 0.9 | (1.1) | 0.7 | (0.6) | 0.228 | 0 | 0.8 | (0.8) | 0.7 | (0.7) | 0.226 | 0 | 0.779 |
| **Other FFQ item** |  |  |  |  |  |  |  |  |  |  |  |  |  |
| Dark bread | 5.1 | (3.9) | 5.3 | (4.3) | 0.748 | 0 | 4.9 | (4.2) | 5.1 | (4.4) | 0.702 | 0 | 0.777 |
| Pizza | 0.5 | (0.4) | 0.5 | (0.8) | 0.815 | 0 | 0.6 | (0.7) | 0.4 | (0.3) | 0.040 | – | 0.162 |
| Hamburgers or hot dogs | 0.4 | (0.3) | 0.4 | (0.7) | 0.642 | 0 | 0.7 | (1.0) | 0.4 | (0.6) | 0.085 | 0 | 0.064 |
| Milk or buttermilk | 10.9 | (4.3) | 9.1 | (5.4) | 0.004 | – | 10.4 | (5.1) | 11.8 | (4.3) | 0.023 | + | <0.001 |
| Cooked vegetables | 2.9 | (3.1) | 2.5 | (3.0) | 0.312 | 0 | 2.3 | (3.3) | 2.3 | (2.6) | 0.969 | 0 | 0.485 |
| Fresh vegetables | 6.3 | (4.3) | 6.3 | (3.7) | 0.968 | 0 | 5.0 | (4.4) | 5.5 | (3.9) | 0.298 | 0 | 0.550 |
| Fruits and berries | 5.7 | (4.0) | 5.0 | (4.1) | 0.149 | 0 | 4.8 | (4.3) | 4.0 | (2.8) | 0.155 | 0 | 0.980 |
| Fresh juice | 3.6 | (4.1) | 2.3 | (2.9) | 0.017 | – | 3.3 | (3.7) | 2.2 | (2.5) | 0.013 | – | 0.666 |
| Salty snacks | 0.8 | (0.7) | 0.7 | (0.6) | 0.578 | 0 | 1.0 | (0.9) | 0.7 | (0.5) | 0.034 | – | 0.245 |

^a^Results from a paired samples t-test.

^b^Results from a two-way mixed ANCOVA (interaction for sex*time), adjusted for age at baseline and follow-up time.

Abbreviations: STI, sweet treat index; FFQ, food frequency questionnaire; SD, standard deviation; WtHR, waist–height ratio.

Supplementary Table 5. Mean (SD) change in weekly consumption frequencies of STI items and other FFQ items among waist gainers (girls and boys whose WtHR was < 0.50 at baseline and ≥ 0.50 at follow-up; n = 199). Missing values for WtHR n = 21.

|  | **Girls (n = 81)** | | | | | | **Boys (n = 118)** | | | | | |  |
| --- | --- | --- | --- | --- | --- | --- | --- | --- | --- | --- | --- | --- | --- |
|  | **Baseline** | | **Follow-up** | |  |  | **Baseline** | | **Follow-up** | |  |  | **Sex*time** |
|  | **Mean (±SD)** | | **Mean (±SD)** | | **p*^a^*** | **Change +/-** | **Mean (±SD)** | | **Mean (±SD)** | | **p*^a^*** | **Change +/-** | **p*^b^*** |
| **STI item** | | |  |  |  |  |  |  |  |  |  |  |  |
| Chocolate and sweets | 1.3 | (1.6) | 1.3 | (0.9) | 0.900 | 0 | 1.5 | (1.2) | 1.6 | (1.3) | 0.336 | 0 | 0.565 |
| Sweet pastries | 0.7 | (0.9) | 0.7 | (0.9) | 0.955 | 0 | 1.2 | (1.6) | 0.9 | (1.0) | 0.017 | – | 0.097 |
| Biscuits/cookies | 1.4 | (1.8) | 0.7 | (0.8) | <0.001 | – | 2.0 | (2.3) | 1.3 | (1.8) | 0.001 | – | 0.935 |
| Sugary juice drinks | 1.5 | (2.5) | 0.9 | (1.4) | 0.053 | 0 | 1.8 | (2.4) | 1.7 | (2.6) | 0.734 | 0 | 0.304 |
| Sugary soft drinks | 1.0 | (1.2) | 0.8 | (0.9) | 0.101 | 0 | 1.6 | (1.9) | 1.3 | (1.2) | 0.214 | 0 | 0.958 |
| Ice cream | 0.8 | (0.9) | 0.6 | (0.5) | 0.093 | 0 | 1.0 | (1.2) | 0.8 | (1.1) | 0.345 | 0 | 0.571 |
| **Other FFQ item** |  |  |  |  |  |  |  |  |  |  |  |  |  |
| Dark bread | 4.6 | (4.4) | 4.4 | (3.6) | 0.707 | 0 | 5.9 | (4.6) | 5.4 | (4.3) | 0.339 | 0 | 0.801 |
| Pizza | 0.7 | (1.3) | 0.4 | (0.4) | 0.091 | 0 | 0.7 | (1.1) | 0.5 | (0.3) | 0.054 | 0 | 0.642 |
| Hamburgers or hot dogs | 0.4 | (0.5) | 0.5 | (0.6) | 0.218 | 0 | 0.6 | (0.7) | 0.5 | (0.5) | 0.787 | 0 | 0.357 |
| Milk or buttermilk | 11.0 | (4.5) | 9.5 | (5.4) | 0.020 | – | 9.6 | (5.5) | 10.7 | (5.1) | 0.011 | + | 0.001 |
| Cooked vegetables | 3.0 | (3.6) | 2.2 | (2.4) | 0.087 | 0 | 2.6 | (3.3) | 2.1 | (2.5) | 0.137 | 0 | 0.445 |
| Fresh vegetables | 6.4 | (4.2) | 7.4 | (4.2) | 0.089 | 0 | 5.0 | (3.8) | 5.8 | (3.9) | 0.040 | + | 0.938 |
| Fruits and berries | 5.9 | (4.5) | 6.0 | (4.7) | 0.888 | 0 | 5.0 | (4.3) | 4.1 | (3.4) | 0.026 | – | 0.216 |
| Fresh juice | 3.6 | (3.9) | 2.4 | (3.0) | 0.018 | – | 3.8 | (3.8) | 2.6 | (3.6) | 0.008 | – | 0.967 |
| Salty snacks | 1.1 | (1.8) | 0.7 | (0.5) | 0.040 | – | 1.1 | (1.0) | 1.0 | (0.7) | 0.324 | 0 | 0.154 |

^a^Results from a paired samples t-test.

^b^Results from a two-way mixed ANCOVA (interaction for sex*time), adjusted for age at baseline and follow-up time.

Abbreviations: STI, sweet treat index; FFQ, food frequency questionnaire; SD, standard deviation; WtHR, waist–height ratio.
